# Supplementary material for: Atomic-precision control of plasmon-induced single-molecule switching in a metal–semiconductor nanojunction
Source: Nat Commun. 2024 Aug 7;15:6709. doi: 10.1038/s41467-024-51000-w (PMC11306799; doi:10.1038/s41467-024-51000-w)
Supplement: Supplementary file 1 — Supplementary Information [file 41467_2024_51000_MOESM1_ESM.pdf]

Supplementary Information: Atomic-Precision Control of Plasmon-Induced Single-Molecule Switching in a Metal–Semiconductor Nanojunction

Youngwook Park[1*], Ikutaro Hamada[2], Adnan Hammud[3], Takashi Kumagai[4], Martin Wolf[1], Akitoshi Shiotari[1*]

[1*]Department of Physical Chemistry, Fritz-Haber Institute of the Max-Planck Society, Faradayweg 4–6, Berlin, 14195, Germany.
[2]Department of Precision Engineering, Graduate School of Engineering, Osaka University, 2-1 Yamadaoka, Suita, Osaka, 565-0871, Japan.
[3]Department of Inorganic Chemistry, Fritz-Haber Institute of the Max-Planck Society, Faradayweg 4–6, Berlin, 14195, Germany.
[4]Institute for Molecular Science, National Institutes of Natural Sciences, 38 Nishigonaka, Myodaichi-cho, Okazaki, Aichi, 444-8585, Japan.

*Corresponding author(s). E-mail(s): park@fhi-berlin.mpg.de; shiotari@fhi-berlin.mpg.de;

Contents

Supplementary Notes **3**

Supplementary Note 1: Calculated adsorption structures 3
Supplementary Note 2: Non-switching molecule at ON state 3
Supplementary Note 3: Reproducibility of the switching event 4
Supplementary Note 4: Zone IV 4
Supplementary Note 5: Lateral tip-position dependence of the switching 5
Supplementary Note 6: Possible mechanisms of the plasmon-driven switch 6
Supplementary Note 7: Simulations of plasmon-enhanced electric field 7

Supplementary Figures **8**

Supplementary Fig. 1: Density functional theory calculated structures of PTCDA, PMI and PDI molecules on Si(111)-7×7 8
Supplementary Fig. 2: Long-lived ON state and its TERS spectra . . . 8
Supplementary Fig. 3: A series of STM-current and TERS measurements during tip approach and retraction at 78 K 8
Supplementary Fig. 4: Control of ON/OFF occupation dynamics by changing gap distance 8
Supplementary Fig. 5: Lateral tip-position dependence of the PTCDA switch 8
Supplementary Fig. 6: Bias dependence of STM images 8
Supplementary Fig. 7: Identification of the reaction mechanism of the plasmon-induced switch 8
Supplementary Fig. 8: Switch activation with λ_{ext} = 633 nm 8
Supplementary Fig. 9: Simulated electric field enhancement in Ag–Ag and Ag–Si junctions 8
Supplementary Fig. 10: Scanning electron microscope images of a Ag tip 8

References **17**

Supplementary Notes

Supplementary Note 1: Calculated adsorption structures

Supplementary Figs. 1a and 1b show the calculated structures of PTCDA/Si(111)-7×7 by the rev-vdW-DF2 and PBE functions, with van der Waals (vdW) interactions considered and ignored, respectively. The latter is in line with the previously reported structure, where the perylene moiety was calculated to be convexly deformed [1]. The adsorption energy of PTCDA/Si(111) was calculated to be -4.21 (-2.63) eV with rev-vdW-DF2 (PBE), which suggests that the vdW interactions between the molecule and the substrate compensate for the large distortion of the C–O–Si bond angle to stabilise the system. When vdW interactions are not evaluated correctly with the calculations, the perylene backbone is deformed to strengthen the O–Si bonds [1], resulting in an underestimation of the stability of the adsorbate.

Supplementary Figs. 1c and 1d show the calculated structures of PMI/Si(111) and PDI/Si(111), respectively, by rev-vdW-DF2. The adsorption geometries of the two molecules are similar in most aspects to that of PTCDA; the molecules are bound to the surface via four O–Si bonds and the molecular plane remains flat.

Supplementary Note 2: Non-switching molecule at ON state

We mainly observed the tip-height dependence for the PTCDA/Si(111) switch as shown in Fig. 2 of the main text; the OFF-state PTCDA molecule in Zone I starts to switch between ON and OFF in Zone II and then the OFF state becomes dominant again in Zone III. In a minor case, instead of returning to the OFF state in Zone III, the molecule remains partially picked up by the tip, having the ON state until the tip height reaches Zone IV (see Supplementary Note 4 for the description of Zone IV). Supplementary Fig. 2 shows the d dependence of the j_{STM} trace and TERS spectrum in the minor case. The discrepancy of the Zone-III behaviour from the typical one (Fig 2 in the main text) probably originates from a coincident atomic structure of the tip apex providing a stronger attractive interaction with the adsorbate, which stabilises the ON state at the tip position.

Under the conditions, we recorded the TERS spectrum of the ON state only (red spectrum of Supplementary Fig. 2c); during the TERS accumulation time (3 s), no switching into the OFF state was detected in the current time trace (Supplementary Fig. 2a). This is the "ON" spectrum shown in Fig. 1c of the main text. The "OFF" spectrum shown in Fig. 1c of the main text was recorded at the tip height before switching started with the same tip.

We note that when the tip becomes closer to the surface (blue arrow in Supplementary Fig. 2a), the TERS intensity of the ON state overall decreases (blue spectrum in Supplementary Fig. 2c and black curve in 2d), even though the ON state occupation remains 1 (purple dotted curve in Supplementary Fig. 2d). This would originate from the decrease in the tilting angle of the lifted molecule, resulting in the change of molecular orientation with respect to the laser polarisation at the junction [2, 3]. Moreover, the resonance-frequency shift and intensity decrease of the plasmon mode at shorter gap distances [4] may also contribute to the overall decrease in the TERS intensity.

Supplementary Note 3: Reproducibility of the switching event

Based on our repeated measurements, we are confident that the switching behaviour is a highly reproducible phenomenon. As statistical evidence, we found out of 115 trials, 101 cases showed switching, i.e, switching occurs nearly every time. Each trial here consists of a sequence of tip approach and retraction on PTCDA under 532 nm irradiation (such as Fig. 2 in the main text or Supplementary Fig. 3), with data collected at both 78 and 10 K. We attribute the 14 non-switching cases (out of 115 trials) to one of the following scenarios, or the combinations of them: (1) the atomic structure of the tip apex being inappropriate for a sufficient attractive interaction with the molecule, (2) stronger molecule–surface bonds that do not dissociate under the same conditions, likely due to atomic-scale defects underneath the molecule that are not discernible with STM images, and/or (3) the tip height not reaching the switchable zone during the approach–retraction tip motion. Importantly, once we found the proper target molecule and tip conditions for switching, we could repeatedly obtain switching features until the junction deformed. As an example, we conducted 9 consecutive measurements (each consisting of a tip approach–retraction set) on the same molecule using the same tip, all of which demonstrated the switching features. This clearly indicates that the switching behaviour is not coincidental.

Supplementary Note 4: Zone IV

Further approach of the tip from Zone III saturates the STM current in the junction. We categorise the tip-height range as Zone IV. Supplementary Fig. 3b shows a j_{STM} plot across Zones I–IV during a tip approach-and-retraction stroke conducted at $T =$ 78 K. Note that unlike the time-resolved trace in Fig. 2 of the main text, the averaged j_{STM} value at each d was shown in this graph, and therefore, the switching in Zone II results in kinks in the j_{STM}–d curve (see the grey ribbon in Supplementary Fig. 3b representing the tunnelling current trend). The j_{STM} saturation at short gap distances suggests the formation of a point contact between the tip and the OFF-state adsorbate ("IV" in Supplementary Fig. 3a). We define the origin of d as the boundary between Zones III and IV, where d indicates the gap distance between the tip apex and the OFF-state adsorbate (see Fig. 2a in the main text and Supplementary Fig. 3a). Supplementary Figs. 3c–f and 3g–j show a series of TERS spectra over Zones during the tip approach (i.e., Zones I to IV) and retraction (Zones IV to I), respectively. The absence (existence) of ν_{PTCDA} peaks in Zones I and III (Zone II) is in good agreement with the dataset at 10 K (Fig. 2 in the main text). In Zone IV, ν_{PTCDA} peaks become visible again (Supplementary Fig. 3f,g), but with one order of magnitude smaller intensity than that in Zone II (Supplementary Fig. 3d,i). This implies that the point-contact configurations are different between the ON state in Zone II and OFF in Zone IV, as schematically shown in Supplementary Fig. 3a. Modifications of TERS intensity of molecules on surfaces were reported as a consequence of changes in molecular orientation [2, 3]. Note that the interpretation of the Raman intensity of the PTCDA/Si switch is more complicated than those in the previous studies; not only the molecular orientation (i.e., tilting angle of PTCDA) but also the dissociation/formation of the O–Si bonds between the molecule and substrate could contribute to the difference between the ON and OFF states.

The results indicate that the TERS peaks of the molecule are detected only when a point contact forms between the tip and the molecule. Although the tip height in Zone III is closer than that in Zone II, no TERS signal was observed in Zone III. This indicates that the chemical enhancement effect is more dominant than the electric field enhancement effect for the Raman scattering intensity of PTCDA on the Si surface.

Supplementary Note 5: Lateral tip-position dependence of the switching

Supplementary Fig. **5** shows the lateral tip-position dependence of the PTCDA/Si(111) switching behaviour. With the tip located either over the molecular edge (Supplementary Fig. **5**a) or over the molecular centre (**5**b), the molecular signals were detected in the TERS spectra together with the j_{STM} jump, which is ascribed to the switching in Zone II. The behaviour at closer tip heights than Zone II depends on the lateral tip position; with the tip over the edge, Zone III did not clearly appear, and Zone II became Zone IV with the tip-height decrease, whereas Zone III appeared with the tip at the same height but over the molecular centre. This is probably because of the differences in the tilting angle and the interaction strength with the tip for the lifted molecule. Note that the appearances of Zones III and IV also depend on the tip apex structure that can be modified by mild poking into a Ag surface or applying voltage pulses. The symmetry and sharpness of the tip apex at the atomic level are expected to determine the Zone-III appearances.

Despite the difference in Zones III and IV, the TERS spectra in Zone II are identical with the two lateral tip positions (see the waterfall plots and Zone-II graphs in Supplementary Fig. **5**). This implies that the structure of the tip–molecule point contact is independent of the lateral tip location. It should be mentioned that the controllable pick-up of a PTCDA molecule by a STM tip has been demonstrated on a Ag(111) surface [5–8]. The previously reported simulation shows that one of the four acyl O atoms is bonded to the tip-apex Ag atom at an O–Ag distance of ~ 2.2 Å, resulting in the molecular pick-up [7]. In contrast, we believe that the ON-state configuration of PTCDA/Si(111) differs from the pick-up structure from the Ag surface for the following reasons. First, in Zone II, the switching of PTCDA/Si(111) was activated by the tip located even over the molecular centre, as described above. In this situation, considering the $\sim 10°$-tilting configuration (as mentioned in the main text), the separation between the tip atom and the oxygen is expected to be more than 7 Å. Second, to pick PTCDA up from Ag(111), the tip atom should attractively interact with the molecule to overcome the interaction between the π electrons of the perylene part and the Ag substrate [5–7]. On the other hand, for PTCDA on the Si surface, the perylene part is separated from the Si atoms due to the adsorption at the corner hole. When the O–Si bonds are ruptured by plasmon, the perylene part can dominantly interact with the Ag tip nearby, stabilising the system.

Supplementary Note 6: Possible mechanisms of the plasmon-driven switch

There are several possible mechanisms for a plasmon-induced chemical reaction at an STM junction [9–12]: direct transition to an excited state (Supplementary Fig. 7a), indirect HC transfer (7b, 1), plasmon-induced vibrational heating (7b, 2) and field-driven (7c) mechanisms.

We ascribed the O–Si dissociation reaction of the anhydride/Si switch to predominantly follow the HC transfer mechanism (Supplementary Fig. 7b, 1; see also Fig. 4f in the main text). This mechanism has been proposed for the dissociation of oxygen molecules chemisorbed on a Ag(110) surface [13, 14], which has the following similarities with the anhydride/Si switch.

First, the O_2/Ag study rules out the contribution of a thermal mechanism since the reaction was triggered at low temperatures (T = 5 K) in a one-photon process. For the anhydride/Si switch, the same switching behaviour was observed at sample temperatures $T = 10$ and 78 K (for example, Fig. 2 in the main text and Supplementary Fig. 3), indicating the T-independent reaction process. Supplementary Fig. 7d shows the laser-power dependence of the O–Si dissociation rate of the anhydride/Si switch, which suggests the linear dependence, as in the O_2/Ag case. This result rules out the predominant contributions of the vibrational heating (multi-photon process; Fig. 7b, 2) or field-driven (nonlinear laser-power dependence; Fig. 7c) mechanism.

Second, both O_2/Ag and anhydride/Si reactions were observed with several λ_{ext} (Figs. 4c and e in the main text), unlike the intramolecular direct-transition mechanism [15, 16]. To support this, we used $\lambda_{\mathrm{ext}} = 633$ nm as a incident laser; the switching was detected in the j_{STM} trace under the laser irradiation (Supplementary Fig. 8a) using an "on-resonant" Ag tip (Supplementary Fig. 8b). Moreover, we measured scanning tunnelling spectroscopy (STS) for PTCDA/Si(111), PMI/Si(111) and bare Si(111) to identify the electronic structures (Supplementary Fig. 7e). We do not find an evident energy matching between the several photon energies of the incident lasers we used and the energy gaps of the adsorbates, suggesting little contribution of the intramolecular or molecule–substrate excitation channel to the reaction.

Finally, in both chemisorption systems, the molecular levels are expected to be sufficiently mixed with that of the substrate (see also the STS in Supplementary Fig. 7e), which effectively opens the plasmon-derived HCs transfer channels into the reactant. These similarities suggest that the switch is HC-transfer-driven.

Under the HC transfer process, a hot carrier entering a molecular level changes the molecular state from the ground state (S_0 in Supplementary Fig. 7b) to a transient charged state (D_0). Note that the D_0 state is intrinsically different from the excited state (S_1), which is involved in the direct transition mechanism (Supplementary Fig. 7a). Because S_0 and D_0 have different equilibrium nuclear distances, the injection of a tunnelling HC into the adsorbate along the reaction coordinate (charging) and its ejection (discharging) provides a kinetic energy to the adsorbate. In the discharging process vibrations of the S_0 state are excited, followed by overcoming the reaction barrier.

Supplementary Note 7: Simulations of plasmon-enhanced electric field

To evaluate the strength of the plasmon-enhanced electric field in the tunnelling junction between a plasmonic Ag tip and a non-plasmonic Si surface, we performed finite element method (FEM) simulations using COMSOL Multiphysics (version 6.1 with the Wave Optics Module).

We used the 3D tip model for a STM junction reported previously (Supporting Information of Ref. [17]): the 300-nm length Ag tip with an effective radius $R = 30$ nm has a half-sphere nanoprotrusion with a radius $r = 0.5$ nm at its apex (see the inset of Supplementary Fig. **9**). A Ag or Si flat plate with a thickness of 100 nm is placed beneath the tip apex with a gap distance z. A p-polarized plane wave with $\lambda_{\text{ext}} = 532$ nm and an electric field $|E_0| = 1$ V/m is incident with an angle of 35° relative to the sample surface. The dielectric constants of the Ag and Si objects are referenced from Refs. [18] and [19], respectively. We set the perfectly matched layers surrounding the volume to absorb all outgoing waves.

At each z, the absolute value of the surface normal component of the electric field $|E_z|$ is sampled at the point 2.5 Å above the sample surface (the red cross in the inset of Supplementary Fig. **9**), which is comparative to the position of the OFF-state molecule on the Si surface based on the DFT calculations (Supplementary Fig. **1**). Note that the Si(111)-7×7 surface is non-flat at the atomic level (with adatoms and corner holes); here we adopted the intermediate height between the first- (*i.e.*, adatoms) and second-layer atom positions as the plate surface in the FEM model, and confirmed that 1-Å variation of the definition does not affect the results qualitatively. Under the conditions, the tip–molecule gap distance d is given by $d = z - 3.2$ Å. Note that quantum effect correction [4] is not considered here, whereas it is required at shorter gap distances ($z < 0.5$ nm).

Supplementary Fig. **9** shows d dependence of the plasmon-enhanced electric field. The enhancement factor of the Ag–Si tunnelling junction at $d = 2$ Å (Zone II) is calculated to be ∼260, whereas the Ag–Ag junction has a more than 4 times higher factor than that. Note that in general TERS intensities are proportional to the fourth power of the electric field [20]; thus, the Raman enhancement over the Si surface is ∼450 times weaker than that over Ag.

Based on the simulation result, we compare the intensity of the plasmon-derived electric field with the direct current (DC) electric field given by V_{bias}. Since $|E_0|$ of the 532-nm incident laser we used in the experiments is $\leq 8.7 \times 10^5$ V/m, the plasmon-enhanced field at the Ag–Si junction is estimated to be $\leq 2.3 \times 10^8$ V/m. This value is exceeded by the DC electric field at $V_{\text{bias}} > 0.12$ V in Zone II. This implies that the plasmon-enhanced electric field is not sufficiently strong to induce a reasonable modification in the potential surface (Fig. **7**c), in line with the above discussion ruling out the field-driven mechanism (Supplementary Note 6). A few volts of V_{bias} provides much larger electric fields in the junction, feasibly destroying the atomic structure of the tip apex (see Fig. 4a and the corresponding discussion in the main text).

Supplementary Figures

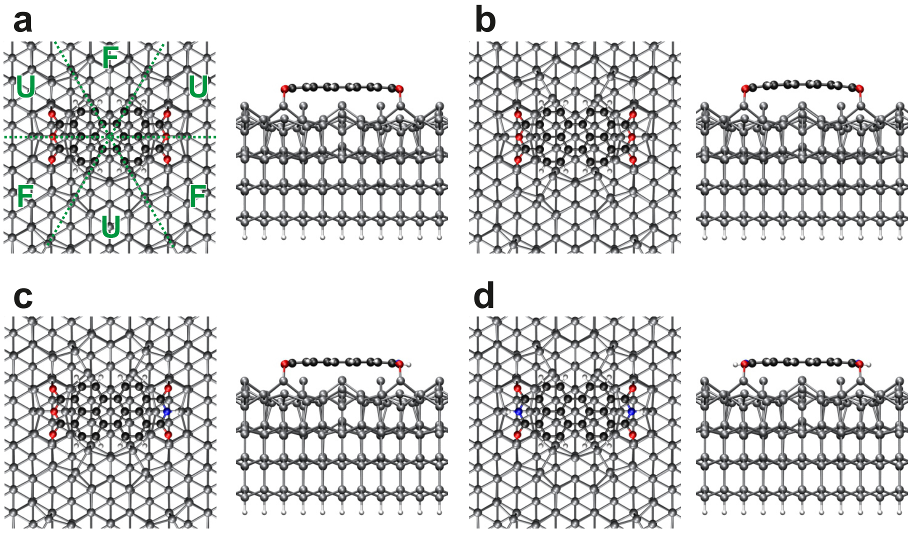

Supplementary Fig. 1 Density functional theory calculated structures of PTCDA, PMI and PDI molecules on Si(111)-7×7. The left (right) panel shows the top- (side-) view. **a**, PTCDA/Si(111) calculated by rev-vdW-DF2. **b**, PTCDA/Si(111) calculated by PBE. **c**, PMI/Si(111) calculated by rev-vdW-DF2. **d**, PDI/Si(111) calculated by rev-vdW-DF2. The white, black, blue, red and grey spheres represent H, C, N, O and Si atoms, respectively. The green dotted lines in **a** represent the boundaries between the half unit cells of Si(111)-7×7. The faulted (unfaulted) half unit cells are labelled as "F" ("U"). Note that the STM images shown in the main text (Figs. 1d, 4a and 4i) are displayed in the same azimuth orientations as the top-view calculated structures.

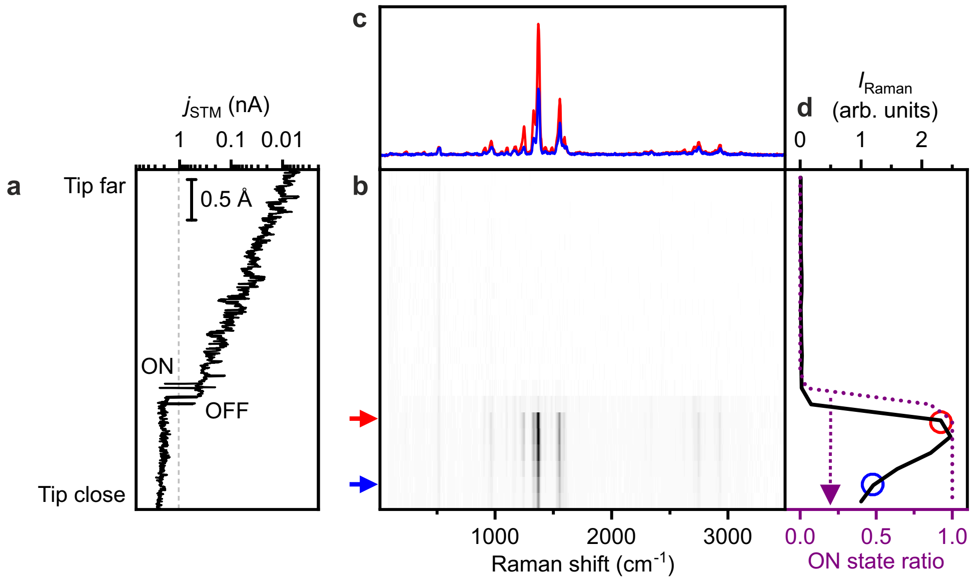

Supplementary Fig. 2 Long-lived ON state and its TERS spectra. **a**, j_{STM} trace during a tip approach over a PTCDA molecule on Si(111)-7×7 (λ_{ext} = 532 nm, P_{ext} = 5.6 mW, V_{bias} = −300 mV, T = 10 K). The maximum ("Tip far" in the graph) and minimum ("Tip close") tip heights are −1.5 and −5.5 Å, respectively, from the STM set point (500 mV and 50 pA). The tip height was reduced by 0.2 Å every 3 s. **b**, A waterfall plot of TERS spectra (white low and black high) simultaneously measured with j_{STM} in **a**. TERS signals were accumulated for the holding time (3 s) at each tip height. The vertical (horizontal) axis represents the tip height (Raman shift). **c**, Representative TERS spectra measured at the tip heights marked with red and blue arrows in **b**. **d**, Line profile of the TERS intensity at 1375 cm^{-1} (black solid curve) and the ON-state occupation ratio calculated from the j_{STM} trace (purple dotted curve).

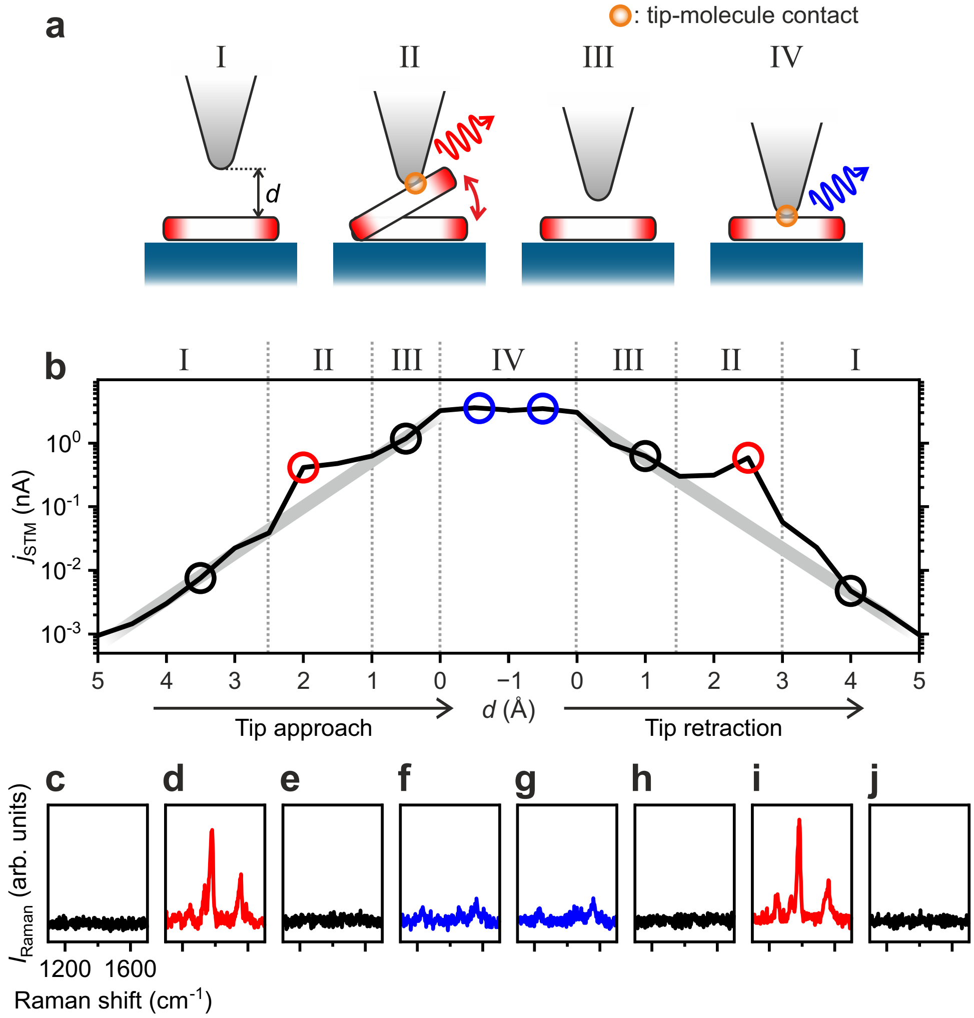

Supplementary Fig. 3 A series of j_{STM} and TERS measurements during tip approach and retraction at 78 K. a, Schematics of a Ag–PTCDA–Si(111) junction in four different gap-distance (d) regions: Zones I–IV. **b**, j_{STM} recorded during a tip approach (left half of the graph) and a subsequent tip retraction (right half) over PTCDA/Si(111) (λ_{ext} = 532 nm, P_{ext} = 5.6 mW, V_{bias} = −400 mV, T = 78 K). The current value averaged for 0.5 s at each d is displayed. The gap distance d changed with a step size of 0.5 Å every 3 s. The grey ribbons indicate an expected j_{STM}–d curve of a tunnelling junction without point-contact formation. Circle markers in the plot correspond to the tip height where the TERS spectra in **c**–**j** were acquired. **c**–**f**, TERS spectra acquired during the tip approach from Zones I to IV (d = 3.5 (**c**), 2.0 (**d**), 0.5 (**e**) and −0.5 Å (**f**)). **g**–**j**, TERS spectra during the tip retraction from Zones IV to I (d = −0.5 (**g**), 1.0 (**h**), 2.5 (**i**) and 4.0 Å (**j**)). All plots in **c**–**j** have identical scale axes.

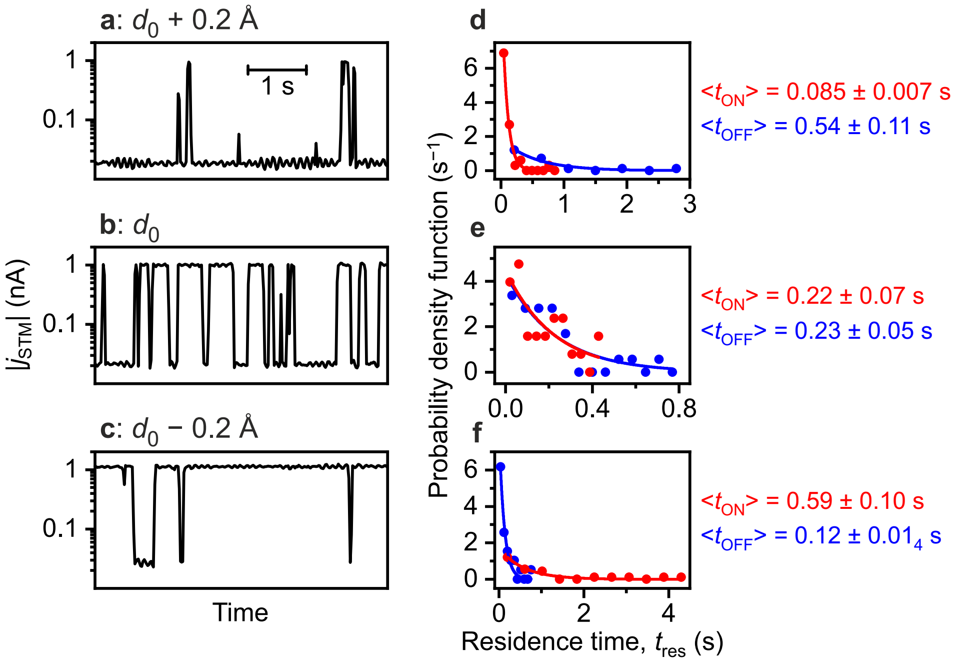

Supplementary Fig. 4 Control of ON/OFF occupation dynamics by changing gap distance. a–c, j_{STM} traces measured at different d over a PMI molecule on Si(111) ($\lambda_{\mathrm{ext}} = 532$ nm, $P_{\mathrm{ext}} = 8$ μW, $V_{\mathrm{bias}} = -300$ mV, $T = 78$ K). The lateral tip position was set to the anhydride side 2 Å away from the molecular centre (the same lateral tip position as in Fig. 3f of the main text). To plot in the log scale, the absolute value $|j_{\mathrm{STM}}|$ is displayed for the current signals at $V_{\mathrm{bias}} < 0$. d in **a** and **c** is larger and smaller, respectively, by 0.2 Å than that in **b** (d_0). The strong d dependence of the ON/OFF occupation of the PMI switch is similar to that of the PTCDA switch shown in the main text (Fig. 2d). **d–f**, Probability density of residence time t_{res} for the ON (red) and OFF (blue) states at the three different d. The statistics of t_{res} was extracted from j_{STM} traces monitored for 30, 10 and 34 s at $d = d_0 + 0.2$ Å, d_0 and $d_0 - 0.2$ Å, respectively. Assuming a Poisson process, the probability density functions are fitted to an exponential distribution, $\frac{1}{\langle t_{\mathrm{res}}\rangle} e^{-\frac{t_{\mathrm{res}}}{\langle t_{\mathrm{res}}\rangle}}$, displayed by the solid curves. The average residence times, $\langle t_{\mathrm{res}}\rangle$, resulting from the fits are shown on the right-hand side of each plot.

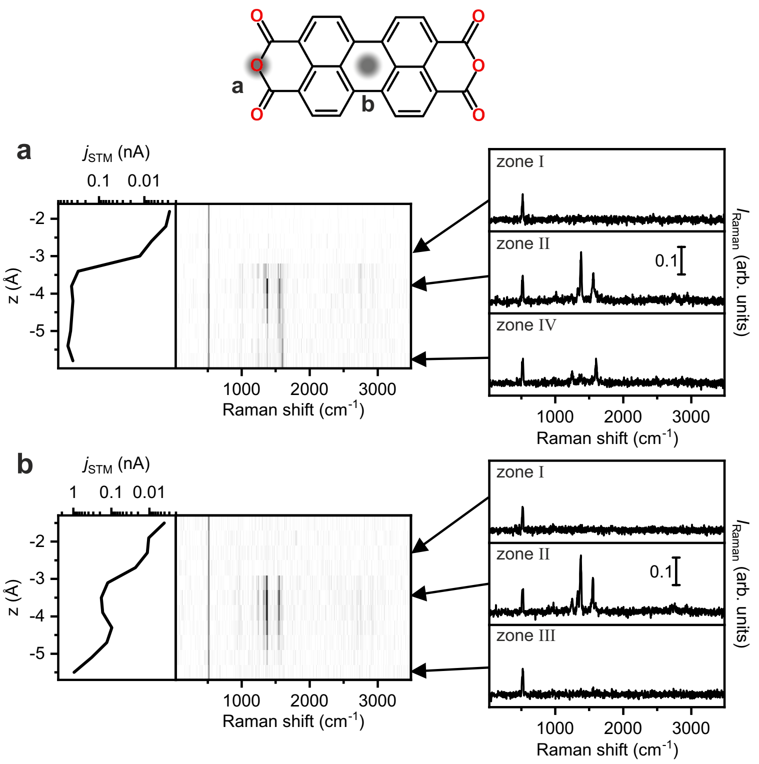

Supplementary Fig. 5 Lateral tip-position dependence of the PTCDA switch. a,b, tip-height z dependence of j_{STM} and TERS spectra with the tip located over the anhydride group and the perylene centre, respectively ($\lambda_{\mathrm{ext}} = 532$ nm, $P_{\mathrm{ext}} = 5.6$ mW, $V_{\mathrm{bias}} = -300$ mV, $T = 78$ K). The left, middle and right panel shows j_{STM} (averaged for 0.5 s at each tip height), a TERS waterfall plot (acquired for 3 s at each tip height) and a representative TERS spectrum in each Zone, respectively. The tip locations for the records in **a** and **b** are labelled on the molecular structure shown in the topmost panel. The origin of z for both tip locations is defined as the STM setpoint height (300 mV and 50 pA) over the molecular centre.

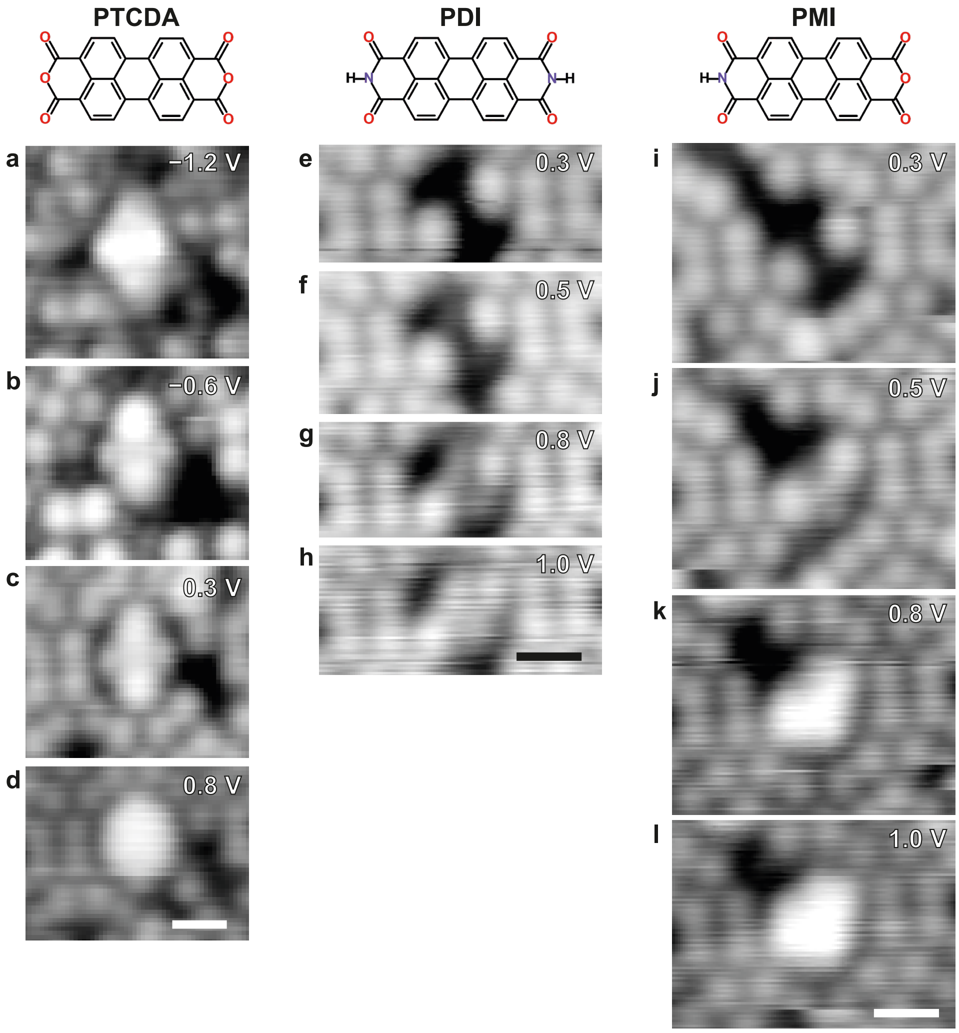

Supplementary Fig. 6 Bias dependence of STM images. **a**–**d**, STM images of a PTCDA molecule on Si(111)-7×7 ($V_{\text{bias}} = -1.2$ (**a**), -0.6 (**b**), 0.3 (**c**) and 0.8 V (**d**)). **e**–**h**, STM images of a PDI molecule on Si(111)-7×7 ($V_{\text{bias}} = 0.3$ (**e**), 0.5 (**f**), 0.8 (**g**) and 1.0 V (**h**)). **i**–**l**, STM images of a PMI molecule on Si(111)-7×7 ($V_{\text{bias}} = 0.3$ (**i**), 0.5 (**j**), 0.8 (**k**) and 1.0 V (**l**)). All the images were obtained in the constant current mode with $j_{\text{STM}} = 50$ pA (Scale bar = 1 nm, $\lambda_{\text{ext}} = 532$ nm, $P_{\text{ext}} = 0.056$ mW, $T = 78$ K). Note that at the range of $P_{\text{ext}} = 0$–5.6 mW at $\lambda_{\text{ext}} = 532$ nm, no laser-power dependence was observed in the STM appearances of the molecules. The bias dependence of PTCDA is in good agreement with those in the literature [21]. At higher positive voltages, the three molecules are observed as bright protrusions. For PMI, the anhydride and imide moieties are clearly discriminable by a STM image at $V_{\text{bias}} \approx 0.5$ V (see also Fig 4a of the main text). In **i**–**l**, the top-left (bottom-right) side of the molecule is ascribed to the imide (anhydride) moiety.

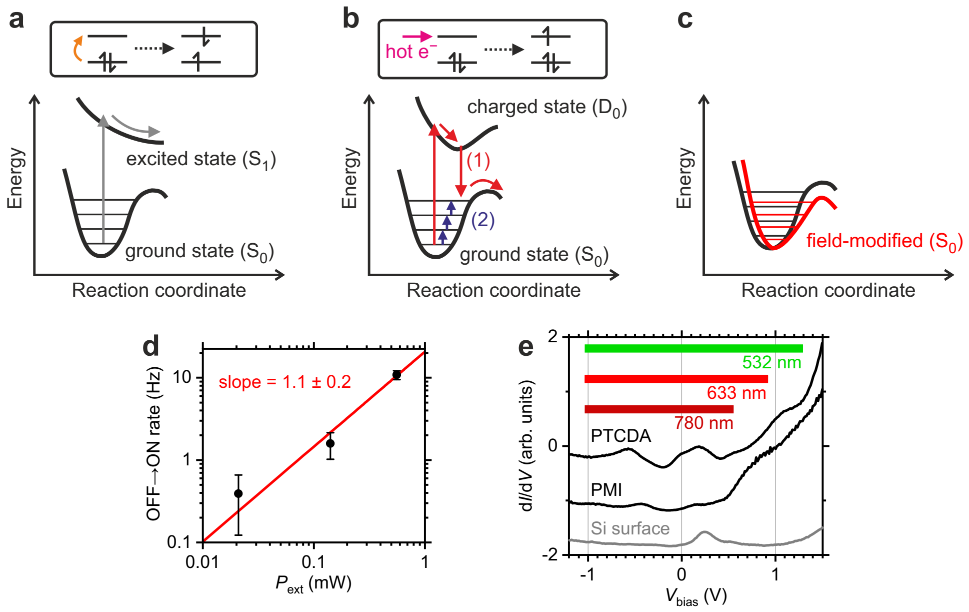

Supplementary Fig. 7 Identification of the reaction mechanism of the plasmon-induced switch. a–c, Potential energy diagrams for the possible pathways of the plasmon-driven single molecule reaction: intramolecular excitation channel (**a**), hot-carrier mediated vibrational excitation channels (**b**) and field-driven deformation of potential surfaces (**c**). The insets on top of the diagrams in **a** and **b** show the change in molecular orbital energy levels during the processes. **d**, OFF-to-ON reaction rate as a function of P_{ext} measured over a PMI molecule (λ_{ext} = 532 nm, V_{bias} = 0 V, T = 78 K). The rates were averaged over j_{STM} traces for 77, 85 and 37 s total duration at P_{ext} = 0.02, 0.14 and 0.56 mW, respectively (error bar: standard deviation). We also confirmed that P_{ext} = 0 gave a rate of 0. The j_{STM} traces were recorded at the onset of Zone II near Zone I by fine tip height adjustment to ensure the identical d at different P_{ext}. The red solid line indicates a linear fit in the double-log plot, resulting in a slope of 1.1 ± 0.2. **e**, STS of PTCDA/Si(111), PMI/Si(111) and bare Si(111). The spectra were recorded by lock-in detection (21 $\mathrm{mV_{rms}}$ at 987 Hz) with the current feedback open (set-point current = 50 pA, V_{bias} = 0.5 V, λ_{ext} = 532 nm, P_{ext} = 8 μW, T = 78 K). The horizontal bars on top of the spectra indicate the photon energy scales of 532 (upper bar), 633 (middle) and 780 nm (lower) as references.

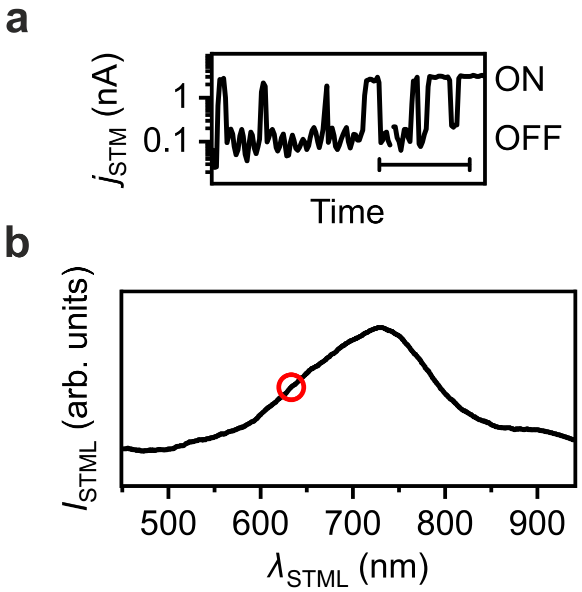

Supplementary Fig. 8 Switch activation with λ_{ext} = 633 nm. a, j_{STM} trace recorded on PTCDA/Si(111) with $\lambda_{ext} = 633$ nm ($P_{ext} = 9.7$ mW, $V_{bias} = -300$ mV, $T = 78$ K, $d = 2.1$ Å, scale bar: 1 s). **b**, STML spectrum recorded over bare Si(111) with the tip used for **a** ($P_{ext} = 0$, $j_{STM} = 1$ nA, $V_{bias} = 3$ V, $T = 78$ K). The red circle indicates the STML intensity at 633 nm.

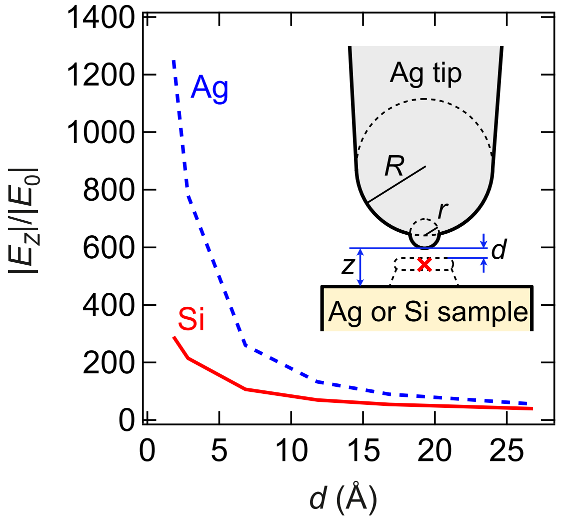

Supplementary Fig. 9 Simulated electric field enhancement in Ag–Ag and Ag–Si junctions. The red solid (blue dotted) curve shows the simulated electric field enhancement at a junction of a Si (Ag) surface and a Ag tip under 532-nm irradiation. The inset shows the side-view scheme of the nanojunction (tip effective radius $R = 30$ nm, nanoprotrusion radius $r = 0.5$ nm). z (d) denotes the gap distance between the sample surface (the OFF-state adsorbate) and the tip apex. In the FEM simulations, the molecule is not considered as a material object but the molecular location on the Si surface is reflected in the sampling point of the electric field, as marked by the red cross.

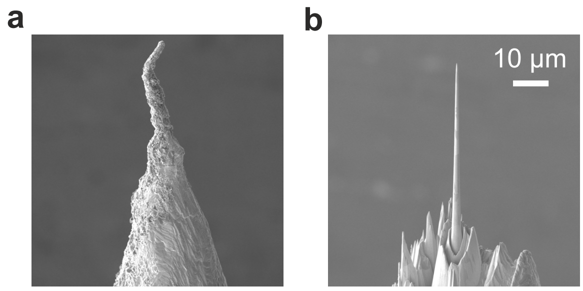

Supplementary Fig. 10 Scanning electron microscope images of a Ag tip. a, An electrochemically etched Ag tip. Note that the tip was probably degraded by long-time exposure under ambient conditions after the etching. **b**, The same tip as in **a** but after being sharpened by the FIB process. We used the sharpened tip for the STM and TERS measurements.

References

[1] Iwata, K., Yamazaki, S., Mutombo, P., Hapala, P., Ondráček, M., Jelínek, P., Sugimoto, Y.: Chemical structure imaging of a single molecule by atomic force microscopy at room temperature. Nat. Commun. **6**, 7766 (2015)

[2] Jiang, N., Chiang, N., Madison, L.R., Pozzi, E.A., Wasielewski, M.R., Seideman, T., Ratner, M.A., Hersam, M.C., Schatz, G.C., Van Duyne, R.P.: Nanoscale chemical imaging of a dynamic molecular phase boundary with ultrahigh vacuum tip-enhanced Raman spectroscopy. Nano Lett. **16**, 3898–3904 (2016)

[3] Cai, Z.-F., Zheng, L.-Q., Zhang, Y., Zenobi, R.: Molecular-scale chemical imaging of the orientation of an on-surface coordination complex by tip-enhanced Raman spectroscopy. J. Am. Chem. Soc. **143**, 12380–12386 (2021)

[4] Esteban, R., Borisov, A.G., Nordlander, P., Aizpurua, J.: Bridging quantum and classical plasmonics with a quantum-corrected model. Nat. Commun. **3**, 825 (2012)

[5] Temirov, R., Lassise, A., Anders, F., Tautz, F.: Kondo effect by controlled cleavage of a single-molecule contact. Nanotechnology **19**, 065401 (2008)

[6] Pump, F., Temirov, R., Neucheva, O., Soubatch, S., Tautz, S., Rohlfing, M., Cuniberti, G.: Quantum transport through stm-lifted single PTCDA molecules. Appl. Phys. A **93**, 335–343 (2008)

[7] Toher, C., Temirov, R., Greuling, A., Pump, F., Kaczmarski, M., Cuniberti, G., Rohlfing, M., Tautz, F.: Electrical transport through a mechanically gated molecular wire. Phys. Rev. B **83**, 155402 (2011)

[8] Brumme, T., Neucheva, O.A., Toher, C., Gutiérrez, R., Weiss, C., Temirov, R., Greuling, A., Kaczmarski, M., Rohlfing, M., Tautz, F.S., Cuniberti, G.: Dynamical bistability of single-molecule junctions: a combined experimental and theoretical study of PTCDA on Ag(111). Phys. Rev. B **84**, 115449 (2011)

[9] Kazuma, E., Jung, J., Ueba, H., Trenary, M., Kim, Y.: STM studies of photochemistry and plasmon chemistry on metal surfaces. Prog. Surf. Sci. **93**, 163–176 (2018)

[10] Wang, M., Wang, T., Ojambati, O.S., Duffin, T.J., Kang, K., Lee, T., Scheer, E., Xiang, D., Nijhuis, C.A.: Plasmonic phenomena in molecular junctions: principles and applications. Nat. Rev. Chem. **6**, 681–704 (2022)

[11] Zhang, Y., Yan, L., Guan, M., Chen, D., Xu, Z., Guo, H., Hu, S., Zhang, S., Liu, X., Guo, Z., Li, S., Meng, S.: Indirect to direct charge transfer transition in plasmon-enabled CO_2 photoreduction. Adv. Sci. **9**, 2102978 (2022)

[12] Zhan, C., Yi, J., Hu, S., Zhang, X.-G., Wu, D.-Y., Tian, Z.-Q.: Plasmon-mediated chemical reactions. Nat. Rev. Methods Primers **3**, 12 (2023)

[13] Kazuma, E., Lee, M., Jung, J., Trenary, M., Kim, Y.: Single-molecule study of a plasmon-induced reaction for a strongly chemisorbed molecule. Angew. Chem. Int. Ed. **59**, 7960–7966 (2020)

[14] Kazuma, E., Lee, M., Jung, J., Trenary, M., Kim, Y.: Real-space observations of multiple reaction pathways enabled by plasmonic hot carriers. J. Phys. Chem. C **127**, 10953–10959 (2023)

[15] Kazuma, E., Jung, J., Ueba, H., Trenary, M., Kim, Y.: Real-space and real-time observation of a plasmon-induced chemical reaction of a single molecule. Science **360**, 521–526 (2018)

[16] Rosławska, A., Kaiser, K., Romeo, M., Devaux, E., Scheurer, F., Berciaud, S., Neuman, T., Schull, G.: Submolecular-scale control of phototautomerization. Nat. Nanotechnol. **19**, 738–743 (2024)

[17] Liu, S., Müller, M., Sun, Y., Hamada, I., Hammud, A., Wolf, M., Kumagai, T.: Resolving the correlation between tip-enhanced resonance Raman scattering and local electronic states with 1 nm resolution. Nano Lett. **19**, 5725–5731 (2019)

[18] Jiang, Y., Pillai, S., Green, M.A.: Realistic silver optical constants for plasmonics. Sci. Rep. **6**, 30605 (2016)

[19] Green, M.A.: Self-consistent optical parameters of intrinsic silicon at 300 K including temperature coefficients. Sol. Energy Mater. Sol. Cells **92**, 1305–1310 (2008)

[20] Pettinger, B., Domke, K.F., Zhang, D., Picardi, G., Schuster, R.: Tip-enhanced raman scattering: influence of the tip-surface geometry on optical resonance and enhancement. Surf. Sci. **603**, 1335–1341 (2009)

[21] Nicoara, N., Paz, Ó., Méndez, J., Baró, A.M., Soler, J.M., Gómez-Rodríguez, J.M.: Adsorption and electronic properties of PTCDA molecules on Si(111)-(7×7): scanning tunneling microscopy and first-principles calculations. Phys. Rev. B **82**, 075402 (2010)
